# Intelligent Biosensors Based on Hyaluronic Acid Hydrogels for Monitoring Chronic Wound Healing with the Involvement of Artificial Intelligence

**DOI:** 10.3390/bios15120773

**Published:** 2025-11-25

**Authors:** Antonia-Mihaela Nicolae, Mihaela Badea, Sandica Bucurica, Florina Rasaliu, Elena Mihaela Constantinescu

**Affiliations:** 1 Department of Fundamental, Prophylactic and Clinical Disciplines, Faculty of Medicine, Transilvania University of Brasov, 56 Nicolae Balcescu, 500019 Brasov, Romania; antonia.nicolae@student.unitbv.ro (A.-M.N.); florina_rasaliu@yahoo.com (F.R.); elena.constantinescu@unitbv.ro (E.M.C.); 2 Research Center for Fundamental Research and Prevention Strategies in Medicine, Research and Development Institute, Transilvania University of Brasov, 10 Institutului St., 500484 Brasov, Romania; 3 Faculty of Medicine, Carol Davila University of Medicine and Pharmacy, Bdul Eroilor Sanitari 8, 050474 Bucharest, Romania; sandica.bucurica@umfcd.ro; 4 Clinical County Hospital of Obstetrics and Gynecology, 36 Gheorghe Bariţiu St., 500025 Brasov, Romania

**Keywords:** chronic wound healing, hyaluronic acid hydrogels, biosensors, artificial intelligence, biomarkers

## Abstract

Chronic wounds, such as those caused by diabetes, burns, and pressure ulcers, pose significant healthcare challenges due to their slow healing and potential for infections. Traditional methods for monitoring wound healing are often intrusive, slow, and lack real-time data. To overcome these limitations, innovative biosensors using hyaluronic acid hydrogels have emerged as a promising solution. As a non-intrusive, biocompatible platform, these biosensors can track pH, glucose levels, inflammatory proteins, and other key biomarkers as wounds heal. With the integration of artificial intelligence (AI), they enable personalized treatment adjustments and early complication detection through real-time data analysis and predictive modeling. This review discusses the recent progress of hyaluronic acid hydrogel biosensors for long-term wound healing, evaluating their strengths, challenges, and potential future improvements. This work aims to enhance chronic wound management and improve multiple clinical outcomes by focusing on the intersection of biomaterial innovation and AI.

## 1. Introduction

Chronic wounds, including diabetic foot ulcers, venous leg ulcers, and pressure ulcers, constitute a significant and growing global healthcare challenge, affecting over 13 million people worldwide and imposing considerable economic and social burdens [1]. Predominantly affecting the elderly, chronic wounds are becoming increasingly widespread and harder to treat, often associated with substantial healthcare expenditures. Recent reports indicate that the five-year mortality rate for individuals with diabetes mellitus and foot ulcers is approximately 40% [2]. These wounds fail to proceed through the normal stages of healing, hemostasis, inflammation, proliferation, and remodeling, resulting in prolonged patient suffering, increased risk of infection, and elevated morbidity and mortality [2,3]. Despite advances in wound care, effective management remains challenging due to the complexity of wound microenvironments, patient comorbidities, and, importantly, the lack of biosensor technologies capable of providing continuous, non-invasive, and reliable monitoring of key biochemical parameters directly at the wound site [3].

Existing wound biosensors face significant limitations: many rely on rigid or semi-rigid substrates that reduce patient comfort and disrupt intimate contact with the wound bed; they often suffer from biofouling and signal instability in the moist, enzyme-rich environment; most detect only limited subset of biomarkers, offering an incomplete picture of wound status; and few can deliver real-time, continuous data without disturbing the tissue [4].

Hydrogels based on natural polymers, particularly hyaluronic acid (HA), have emerged as promising materials for wound dressings due to their excellent biocompatibility, hydrophilicity, and ability to mimic the native extracellular matrix (ECM) [3]. Hyaluronic acid plays a crucial biological role in tissue hydration, cell migration, and the modulation of inflammation, can encapsulate sensing elements, protect them from degradation, and allow selective diffusion of analytes such as pH, glucose, and inflammatory mediators, making it an ideal component in biosensors designed to monitor wound biomarkers such as pH, glucose, and inflammatory mediators [3]. Incorporating HA hydrogels into sensor platforms facilitates the continuous and non-invasive assessment of wound status while maintaining a bioactive environment conducive to healing.

Recent developments have focused on integrating intelligent sensing capabilities with HA-based hydrogels to create multifunctional wound dressings capable of real-time biomarker detection and therapeutic intervention [4]. These biosensors often combine optical and electrochemical detection methods, enabling precise quantification of critical analytes at physiologically relevant concentrations [5]. However, persistent challenges include achieving long-term stability in the dynamic wound microenvironment, enabling simultaneous detection of multiple biomarkers, and maintaining optimal mechanical properties for patient comfort and sensor durability.

Artificial intelligence (AI) has recently been introduced as a powerful tool to enhance data analysis from these biosensors, offering advanced algorithms for pattern recognition, predictive modeling, and personalized wound management [6]. Machine learning and deep learning techniques can interpret high-dimensional, time-series data generated by sensors to distinguish between normal and pathological healing trajectories, potentially enabling earlier clinical interventions [6]. Nonetheless, the integration of AI in wound care also requires addressing challenges related to data standardization, quality assurance, patient privacy, and compliance with regulatory frameworks.

This review critically evaluates the current state of HA hydrogel-based biosensors for chronic wound monitoring, emphasizing how HA materials specifically address key gaps in existing biosensor technologies. It further explores AI’s role in improving biosensor data interpretation and discusses future directions for developing fully integrated, intelligent wound care systems. Our objective is to provide a comprehensive overview that supports the design of next-generation smart dressings capable of improving healing outcomes and patient quality of life. All articles included met the criteria: experimental or clinical application of HA-based hydrogel biosensors; monitoring at least one biomarker (pH, glucose, inflammatory proteins); direct integration of HA into the sensor matrix; and sufficiently detailed detection techniques for reproducibility [3,6].

## 2. Chronic Wound

Wound healing is a complex physiological process that occurs in four overlapping phases: hemostasis, inflammation, proliferation, and remodeling. The hemostatic phase begins immediately after injury, with vasoconstriction and blood clotting, which prevents blood loss and forms a provisional matrix for cell migration. The inflammatory phase lasts up to seven days, during which neutrophils and macrophages clear the wound and release growth factors. The proliferative phase follows, characterized by granulation tissue formation, new blood vessel growth, and epithelialization. The final remodeling phase can last up to two years, during which collagen is organized and strengthened [7].

**Figure 1:**
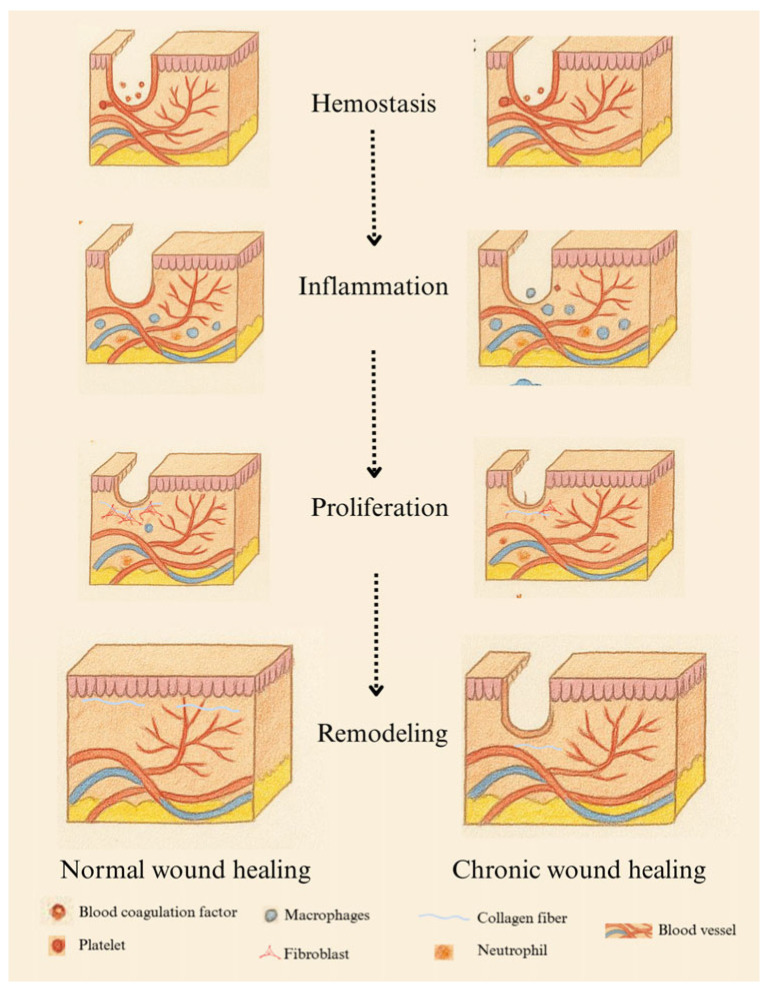
Comparison of the healing process between a chronic wound and a normal wound. In the wound area, a U-shaped red line denotes the hemostatic stage, while a yellow line indicates stalled healing and ongoing inflammation. The blue line represents collagen fiber deposition during the proliferative and remodeling phases. The dashed vertical arrow indicates the chronological progression through the four wound-healing stages.

Chronic wounds fail to progress through normal healing stages, often getting stuck in the inflammatory phase (Figure 1). They exhibit high levels of pro-inflammatory cytokines, proteases, and reactive oxygen species (ROS), along with persistent infections and dysfunctional stem cells. Excessive protease activity degrades the extracellular matrix (ECM), preventing transition to the proliferative phase. Additionally, oxidative stress increases ROS levels, which damage ECM proteins and worsen inflammation [7,8]. Research suggests that potent antioxidants can mitigate ROS levels and support the healing process.

Another major issue in chronic wounds is the presence of senescent cells, which have impaired proliferative and secretory functions, making them unresponsive to normal healing signals. Fibroblasts, keratinocytes, endothelial cells, and macrophages in chronic wounds often exhibit senescence, contributing to delayed healing. This dysfunction is linked to oxidative stress and metabolic abnormalities, particularly in diabetic patients [8,9].

**Figure 2:**
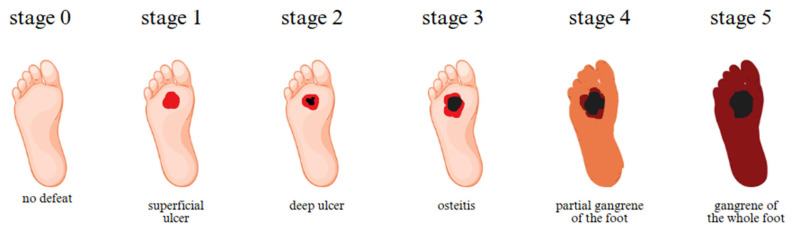
Diabetic foot ulcer stages.

Mesenchymal stem cells (MSCs) play an essential role in wound healing, but in patients with chronic wounds or diabetes, these cells are often deficient or defective (Figure 2). Direct delivery of functional donor-derived MSCs may help restore healing capacity [10].

In Figure 2, the progressive stages of diabetic foot ulcer development are illustrated, starting from stage 0 (no open lesions or healed lesions) to stage 5 (complete gangrene of the foot). Stage 1 represents a superficial ulcer, not penetrating into deeper tissue layers; stage 2 shows a deep ulcer extending to the tendon, bone, or joint; stage 3 depicts deep ulcers with infected tissues, also referred to as osteitis; stage 4 indicates partial gangrene of the foot; and stage 5 corresponds to gangrene affecting the entire foot.

Proper management of chronic wounds requires addressing underlying systemic issues such as infection or vascular disease. Effective treatment begins with a thorough patient and wound assessment, followed by targeted interventions to remove contributing factors. Wound bed preparation is essential to creating an optimal healing environment. Accurate diagnosis is fundamental for determining the wound’s etiology, whether it be diabetic foot ulcers (DFUs), venous leg ulcers (VLUs), pressure ulcers (PUs), post-surgical wounds, or atypical wounds like pyoderma gangrenosum. Clinicians must also rule out malignancy, particularly in cases of squamous cell carcinoma [11,12].

## 3. Hyaluronic Acid

Hyaluronic acid (HA) is a biocompatible and biodegradable polysaccharide, widely studied for its involvement in tissue regeneration, cellular signaling, and water retention. Thanks to its viscoelastic properties and ability to form hydrogels, HA represents a promising material for the development of smart biosensors [13]. In the context of chronic wound monitoring, HA-based hydrogels offer a favorable environment for embedding sensing components that can track biological markers in real time. To be effective in wound-healing applications, HA hydrogels must possess high biocompatibility, suitable mechanical strength, and conformability to irregular wound surfaces. They should demonstrate controlled swelling, maintain a balanced moisture level, and adhere gently to tissue without causing trauma during removal. Resistance to premature enzymatic degradation and compatibility with integrated biosensing elements are also essential for long-term functionality [14,15].

### 3.1. Physicochemical Properties

**Figure 3:**
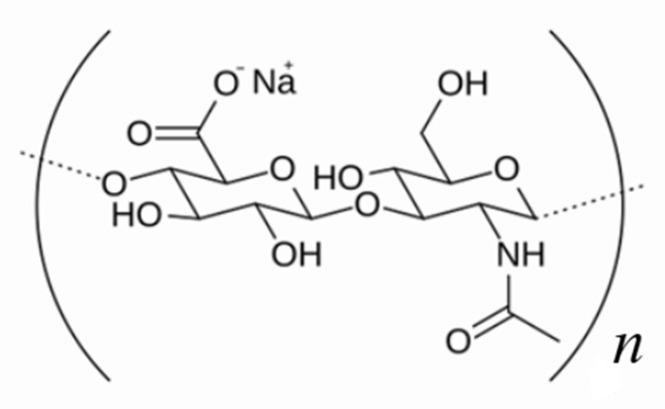
Sodium hyaluronate structure.

In aqueous solution, hyaluronic acid adopts an extended, random coil-like structure, stabilized by hydrogen bonds (Figure 3). This configuration is essential for the rheological behavior of HA. The hydrogen bonds are aligned parallel to the polymer chain axis and are essential for maintaining the structural stability of the molecule [16]. In dilute solutions, hyaluronic acid forms a three-dimensional polymer network; however, at low concentration conditions, these chains do not interact with each other, and the solution remains viscoelastic and pseudoplastic. This characteristic makes HA have a non-Newtonian behavior, with a viscosity that decreases as shear increases [17].

The rheological properties of hyaluronic acid are significantly influenced by physicochemical factors, such as molecular weight, concentration, pH and solution temperature. At higher concentrations, hyaluronic acid forms interconnected networks, and interactions between polymer chains lead to an increase in viscosity [17].

In addition, the viscosity of HA solutions decreases significantly under conditions of elevated temperature and alkaline pH, suggesting a weakening of interactions between polymer chains. For example, at pH conditions higher than 11, hyaluronic acid undergoes significant degradation, and the hydrogen bonds that maintain the stability of the molecular structure are broken, thus affecting the integrity of the polymer network. In acidic environments (pH < 4), HA degradation is slower, but can continue through hydrolysis mechanisms [16].

Ionic strength is also another property that influences the rheological behavior of HA. At high salt concentrations, the electrostatic interactions between the carboxylate groups of hyaluronic acid are modified, which leads to a decrease in viscosity and a reduction in the ability to form stable networks [17]. At the same time, hyaluronic acid is extremely sensitive to temperature changes, and at low temperatures, the polymer chains tend to form more compact structures, which leads to an increase in the viscosity of the solution [16].

Hyaluronic acid solutions are non-thixotropic: as the shear rate decreases and stops, they recover their original structure and viscosity by following the same intermediate states of the breakdown process. Therefore, the polymeric network breakdown is transient and reversible, allowing easy application, good mechanical resistance, and rapid adaptation to environmental changes [15].

Another important aspect is the pseudoplastic behavior of hyaluronic acid, which causes HA solutions to reduce their viscosity under the action of an external force (such as shear) and recover the initial viscosity when the force is removed [15].

Changes in pH and ionic strength are determining factors in the behavior of hyaluronic acid. In alkaline solutions (pH > 9), hyaluronic acid undergoes significant structural changes due to the breaking of hydrogen bonds, leading to reduced viscosity and polymer degradation. Also, in concentrated saline solutions, the rheological behavior of HA is modified by increasing ionic strength, which reduces the electrostatic effect between the polymer chains and allows them to relax, creating a less viscous structure [17].

The hydrogel must be strong enough to support the integrated electronics without tearing during application or use, yet soft and flexible enough to conform to wound contours. Viscosity is particularly important for injectable or moldable hydrogels, as it affects both ease of application and the diffusion of wound exudate to the sensing sites [18].

The functional lifetime of an HA hydrogel-based AI system depends on both the hydrogel’s stability and the electronics’ power source. Hydrogel formulations can be tuned to last from several days to weeks by adjusting crosslink density, while low-power AI chips can operate within similar timeframes. In clinical use, dressing change protocols, typically every 3 to 7 days, often define the maximum duration of continuous monitoring, though extended use is possible in controlled environments [18].

### 3.2. Synthesis

**Figure 4:**
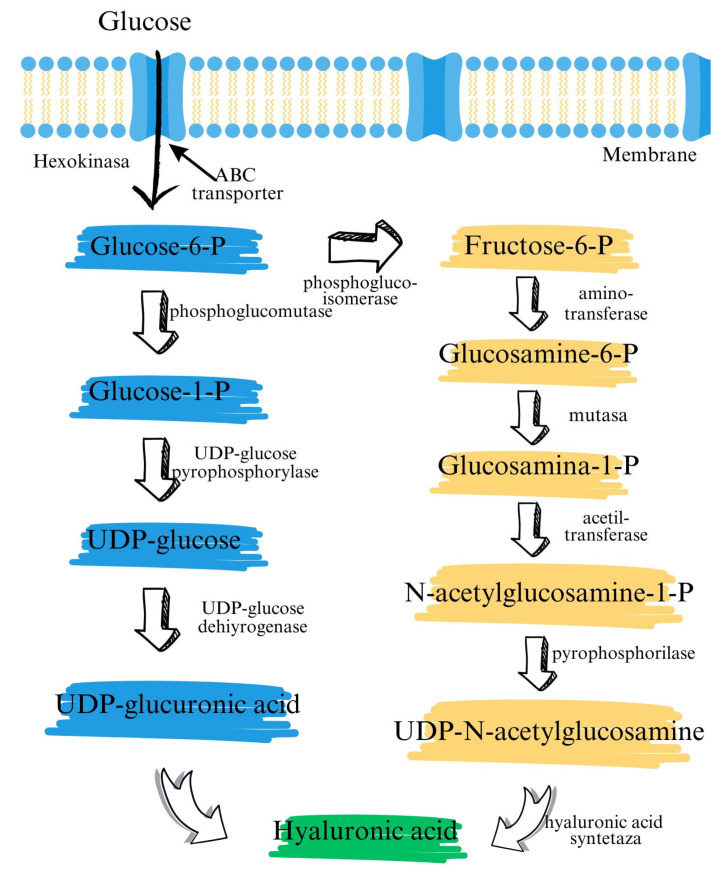
Hyaluronic acid synthesis.

Unlike most glycosaminoglycans, which are produced in the Golgi apparatus and then secreted into the extracellular space, hyaluronic acid is synthesized directly at the plasma membrane by a class of enzymes called hyaluronan synthetases [19]. Secondary structure predictions and homology modeling indicate an integral membrane protein (IMP). An integral membrane protein is a protein molecule (or set of proteins) that, in most cases, spans the biological membrane with which it is associated (especially the plasma membrane) or that is sufficiently embedded in the membrane to remain attached to it during the initial stages of biochemical purification (as opposed to peripheral membrane proteins). Hyaluronan synthase enzymes synthesize large, linear polymers of the repeating disaccharide structure of hyaluronan by the alternative addition of glucuronic acid and N-acetylglucosamine to the growing chain, using their activated nucleotide sugars (UDP-glucuronic acid and UDP-N-acetylglucosamine) as substrates (Figure 4) [19].

In the skin, hyaluronic acid is synthesized mainly by dermal fibroblasts and epidermal keratinocytes. In fibroblast cultures, the rate of hyaluronic acid biosynthesis is regulated in part by cell density. At low cell densities, biosynthesis is high, and cell motility and proliferation are also high. At high cell densities, cell proliferation is low, and hyaluronic acid biosynthesis is stopped [19,20].

### 3.3. Hyaluronic Acid in Wound Healing

Hyaluronic acid exhibits distinct biological functions depending on its molecular size. High-molecular-weight HA molecules contribute to maintaining the hydration and structure of the extracellular matrix, exerting a protective effect on the cells involved in the healing process, while smaller fragments promote cell migration, facilitating the recruitment of fibroblasts and keratinocytes to the injury site. Intermediate-sized HA chains stimulate the expression of inflammatory cytokines, thus accelerating the inflammatory process necessary for healing, while very short oligomers, comprising as few as four saccharide units, induce chemotaxis, attracting immune cells required for debris clearance and infection control [13].

During the inflammatory phase, hyaluronic acid synthesis increases rapidly. Large molecules of hyaluronic acid, derived from platelets and transported through the bloodstream, reach the site of injury, where they bind to fibrinogen, thus initiating the extrinsic coagulation process. At the same time, edema generated by the accumulation of hyaluronic acid contributes to the swelling of the tissues around the wound. This phenomenon is essential for cell migration to facilitate the creation of a hydrated environment that is easy for new connective tissue [21].

In the proliferative phase, hyaluronic acid supports fibroblast proliferation and collagen synthesis, essential for the formation of new connective tissue. It also stimulates angiogenesis by interacting with the CD44 (Cluster of Differentiation 44) receptors of endothelial cells, which leads to the formation of new blood vessels necessary for the oxygenation and nutrition of the regenerated tissue [13,21].

During the remodeling phase, hyaluronic acid, by acting on the RHAMM (Receptor for Hyaluronan-Mediated Motility) receptor, facilitates the reorganization of the cellular cytoskeleton and the stimulation of regeneration mechanisms by activating the ERK1/2 (Extracellular Signal-Regulated Kinases 1/2) and FAK (Focal Adhesion Kinase) signaling pathways, which are involved in cell survival and migration [13]. In addition, the interaction with ICAM-1 (Intercellular Adhesion Molecule-1) plays an essential role in the immune response, limiting the recruitment and activation of leukocytes (white blood cells) at the level of joints affected by inflammation [13,22]. The gradual degradation of hyaluronic acid, by the action of hyaluronidases, also ensures the balance between synthesis and degradation, optimizing the quality of the newly formed tissue [13,22].

In terms of pain modulation, hyaluronic acid acts as an inhibitor of the transmission of nociceptive impulses (pain signals), interacting with ion channels and receptors involved in pain perception, such as TRPV1 (Transient Receptor Potential Vanilloid 1) and ASIC3 (Acid-Sensing Ion Channel 3), decreasing sensitivity to painful stimuli [22].

Due to its multifunctional biological properties, hyaluronic acid can thus be used in modern wound healing treatments, including bioactive dressings, gels and regenerative therapies. Studies show that exogenous application of hyaluronic acid can accelerate the healing process, reduce inflammation and prevent the formation of hypertrophic scars [23].

## 4. Principles of Hyaluronic Acid-Based Biosensors

The versatility of HA allows for functionalization with conductive nanomaterials such as graphene and gold nanoparticles, as well as bioactive molecules, enhancing its applicability in biosensors. Through chemical modifications such as cross-linking or conjugation with functional groups, HA hydrogels can be tailored to achieve selective analyte detection and improved mechanical stability [3,24]. These modifications also enhance sensor performance for real-time monitoring: the hydrated, porous HA network facilitates rapid diffusion of biomarkers to the sensing elements, improving sensitivity, while its viscoelasticity and semi-permeable structure provide mechanical and chemical stability, ensuring consistent signal acquisition even in dynamic or enzyme-rich wound environments. Functionalization with conductive elements enhances HA’s electrical properties, allowing it to be used in electrochemical biosensors for precise biomarker detection. Additionally, HA-based nanocomposites improve sensor sensitivity and durability, ensuring long-term functionality in chronic wound monitoring by meeting key needs such as: continuous, real-time data acquisition without disturbing the wound bed, reliable multi-biomarker detection in moist, enzyme-rich environments, high biocompatibility to avoid irritation or rejection, and long-term sensor stability to reduce dressing changes [5].

Hyaluronic acid-based biosensors integrate biocompatible hydrogel matrices with advanced sensing mechanisms to enable real-time monitoring of chronic wound healing. The intrinsic hydration and semi-permeable network of HA allow efficient interaction with wound exudates and rapid biomarker transport, which enhances the responsiveness of embedded sensors. At the same time, the mechanical resilience of HA maintains structural integrity during prolonged monitoring, contributing to sensor stability. These platforms address the actual clinical requirements of chronic wound monitoring, specifically, early infection detection, accurate tracking of healing phases, differentiation between transient and pathological inflammatory responses, and seamless integration with predictive analytics tools, thus supporting timely and personalized treatment decisions. These biosensors detect critical biomarkers such as pH, glucose, inflammatory proteins, and temperature variations, offering valuable insights into wound healing. The core principle of their operation is the ability of hyaluronic acid (HA) to form a hydrated, semi-permeable matrix that interacts with wound exudates, allowing for efficient diffusion of target molecules to embedded sensing elements [6]. Additionally, based on the information gathered from these sensors, the release of HA can be triggered, further enhancing the wound healing environment by providing necessary moisture and promoting tissue repair.

The biosensors embedded within HA hydrogels typically operate through optical or electrochemical detection methods. Optical sensors rely on fluorescence, colorimetric changes, or luminescence in response to variations in biomarker concentrations. These biosensors provide a non-invasive and real-time assessment of the wound environment, allowing for visual tracking of healing progress. The fluorescence-based sensors, for example, exhibit intensity changes in response to pH fluctuations, indicating potential infection or tissue regeneration status [25]. Electrochemical sensors detect biomolecular interactions within the hydrogel matrix by measuring electrical properties such as impedance, current, or potential differences. The changes in these parameters reflect variations in biomarker levels, enabling precise and quantitative wound assessment. Such sensors can be miniaturized and embedded within flexible hydrogel structures for enhanced biocompatibility and real-time monitoring [25]. The combination of HA’s conductivity, porosity, and mechanical stability with these sensing methods is what allows accurate, continuous monitoring without sensor degradation or signal loss.

The integration of an AI-based chip into a hyaluronic acid (HA) hydrogel wound dressing can be achieved through flexible, biocompatible electronic designs. These may include stretchable circuit patterns printed on elastomeric substrates or “island–bridge” microchip layouts embedded within the hydrogel layer. The sensor components are positioned at the hydrogel–wound interface to collect data, while the AI-enabled chip, either embedded or connected wirelessly, processes the information in real time, enabling predictive analytics and personalized feedback for wound management [26,27].

We summarized the main experimental findings reported in the literature regarding hyaluronic acid-based hydrogel biosensors used for chronic wound monitoring. Key features include sensor design, biomarker detection capabilities, functional performance, and artificial intelligence (AI) integration.

### 4.1. Optical Biosensors

**Figure 5:**
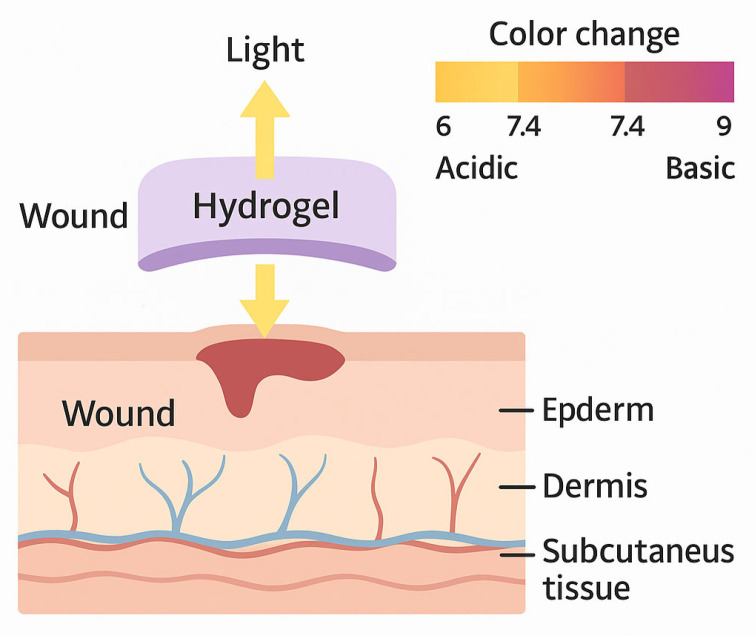
Schematic representation of a light-activated hydrogel biosensor for wound pH monitoring.

Healthy skin exhibits a slightly acidic pH between 4 and 6, which inhibits bacterial colonization. Upon injury, the pH rises towards neutrality (around 7.4), creating a conducive environment for bacterial proliferation [2]. Chronic wounds typically exhibit a more alkaline pH range of 7 to 9, correlating with an increased risk of infection and impaired healing. Hyaluronic acid (HA)-based hydrogels functionalised with pH-sensitive dyes such as phenol red or hydroxyl-substituted azobenzene provide a biocompatible and highly swollen matrix capable of real-time pH monitoring [5]. These optical sensors detect pH changes across a physiologically relevant range (5–10) through visible colorimetric shifts, enabling non-invasive wound status visualization (Figure 5). Their swelling ratio can reach up to 400%, enhancing dye retention and sensor stability during prolonged wound application. The response time is rapid, typically under 2 min, making them suitable for continuous wound monitoring [2]. One disadvantage of using optical biosensors based on hyaluronic acid is their limited stability under varying environmental conditions, which can affect sensor performance and accuracy over time.

### 4.2. Electrochemical Biosensors

**Figure 6:**
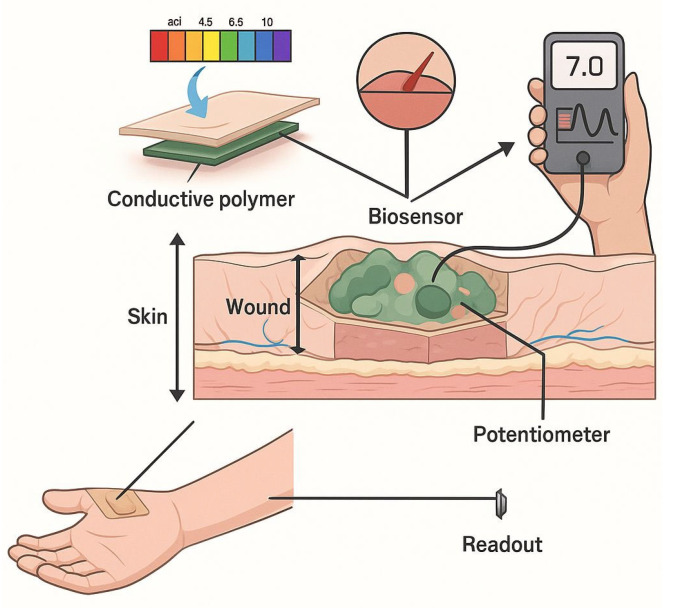
Schematic illustration of an electrochemical biosensor for wound pH monitoring .

Complementing optical methods, electrochemical pH sensors integrated into HA hydrogels utilize conductive polymers such as polyaniline (PANI), achieving Nernstian sensitivity (~59 mV/pH) across pH 4–9. These sensors, fabricated via screen-printing techniques with carbon and Ag/AgCl electrodes, offer high reproducibility and mechanical flexibility, making them suitable for wearable wound dressings (Figure 6). Response times are generally under 30 s, enabling precise, real-time pH tracking critical for early infection detection and personalized treatment adjustment [2,5].

Wearable electrochemical pH sensors offer a more precise method for monitoring wound pH by measuring electrical potential and current. Early glass-electrode-based sensors measured electrical potential, while inorganic electrochemical sensors were later developed using materials like metals (Pd, Bi, Sb), metal oxides (TiO_2_, ZnO), and others (Si_3_N_4_, InAs). For wound monitoring, flexible pH-sensitive polymers, including conductive (polyaniline—PANI), semiconducting (polythiophenes), and insulating (parylene) materials, were also created [5].

Although hyaluronic acid (HA) provides advantages such as biocompatibility and resistance to biofouling in electrochemical biosensors, it also presents drawbacks like low electrical conductivity and possible instability under specific conditions. Additional limitations include the requirement for specialized stabilization methods to prevent aggregation, a potentially narrow strain-sensing range, and difficulties in scaling up production.

## 5. Biomarker Detection Performance Using HA Hydrogels

**Figure 7:**
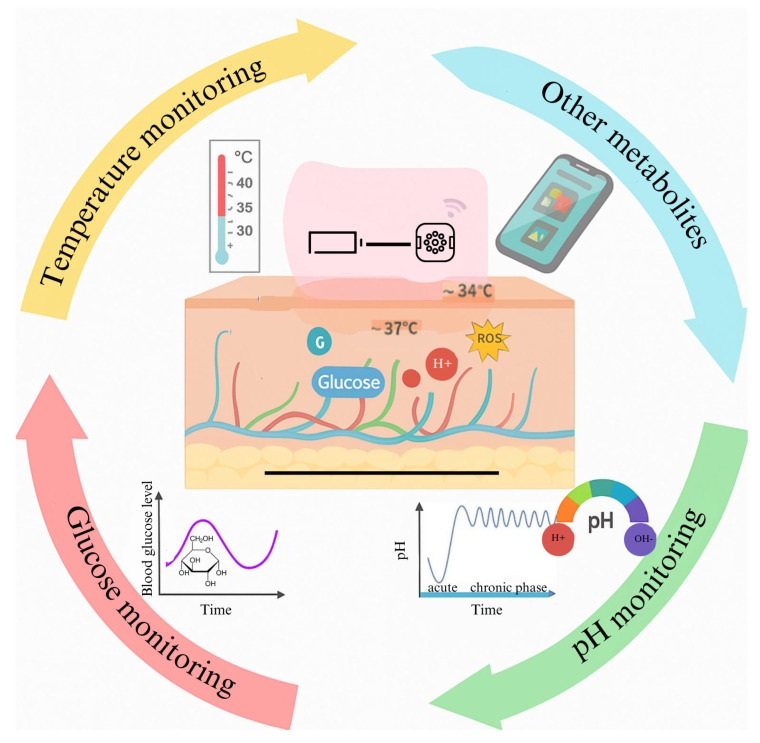
An illustration of wearable wound sensors for real-time monitoring of wound status.

Altered biomarker levels in CW indicate disruptions in the healing process, making biomarkers essential for wound detection, diagnosis, and ongoing monitoring (Figure 7) (Table 1). Their concentrations provide critical clinical insights into wound progression and treatment response, enhancing the precision and accuracy of wound care practices [5].

### 5.1. Glucose Monitoring

Glucose concentration monitoring is another key parameter in chronic wounds, especially diabetic ulcers, where hyperglycemia disrupts normal healing. Typical glucose levels in wound exudate range from 0.1 mM in healthy conditions to over 20 mM in poorly controlled diabetic wounds. Enzyme-based electrochemical sensors using glucose oxidase (GOx) immobilized within HA hydrogels catalyze glucose oxidation to gluconic acid and hydrogen peroxide, the latter using amperometric detection [3]. These sensors demonstrate high sensitivity with detection limits as low as 5 µM, linear response up to 30 mM glucose, and rapid response times (<5 min). Nanocomposite enhancements with carbon nanotubes or graphene embedded in the hydrogel matrix significantly improve electron transfer efficiency, reducing detection limits to approximately 1 µM and enhancing sensor stability [3,28,29].

Optical glucose sensors integrated into HA hydrogels utilize fluorescent boronic acid derivatives that reversibly bind glucose, inducing measurable fluorescence intensity shifts. When incorporated into hydrogels, fluorescent glucose-binding proteins provide a noninvasive means of monitoring glucose levels through changes in fluorescence intensity or wavelength [28]. These probes operate effectively in the 0.1–25 mM range, allowing for real-time, non-invasive glucose monitoring without electrochemical interference, thus complementing enzyme-based methods and enabling multimodal sensing platforms for diabetic wound care [28,29]. This method allows for real-time visual monitoring of glucose, providing an immediate and intuitive readout of the biochemical status of the wound. Optical sensors have the advantage of being less susceptible to interference from other electroactive substances that may be present in the wound exudate. When incorporated into HA hydrogels, these sensors complement electrochemical readouts, ensuring that glucose fluctuations—critical for adjusting treatment in diabetic patients—are reliably detected [5].

A dual-sensing platform integrates electrochemical and enzyme-based optical glucose sensors into HA-based hydrogels, improving the reliability and accuracy of glucose monitoring in diabetic wounds. This multimodal approach not only provides continuous and real-time data but also supports personalized treatment adjustments, ultimately leading to improved wound healing outcomes and a reduced risk of infection [5].

### 5.2. Reactive Oxygen Species (ROS)

Reactive oxygen species (ROS), particularly hydrogen peroxide (H_2_O_2_), serve as additional biomarkers due to their dual role in microbial killing and tissue damage. Chronic wounds often present elevated ROS levels ranging from nanomolar to micromolar concentrations. HA hydrogels functionalised with electrochemical sensors based on Prussian Blue-modified electrodes or fluorescent probes exhibit sensitive detection of H_2_O_2_ down to 100 nM with real-time monitoring capability. This sensitivity allows for early detection of oxidative stress imbalance, guiding antioxidant therapies and improving healing outcomes [5,28].

### 5.3. Protease Monitoring (MMP-9)

**Figure 8:**
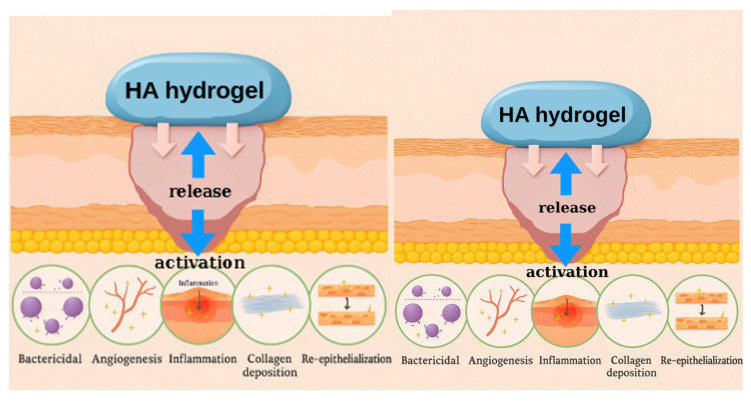
Mechanism of action for HA hydrogel as a wound healing biomaterial.

Matrix metalloproteinase-9 (MMP-9), a protease elevated in chronic wounds, degrades extracellular matrix components, hindering tissue regeneration. MMP-9 concentrations in wound fluid typically vary from 10 ng/mL to several micrograms per milliliter. HA hydrogels integrated with peptide-based MMP-sensitive fluorogenic substrates or electrochemical sensors provide specific detection with limits as low as 5 ng/mL. This allows for precise proteolytic activity and wound severity assessment, facilitating tailored therapeutic interventions (Figure 8) [24,28].

### 5.4. Inflammatory Proteins

**Figure 9:**
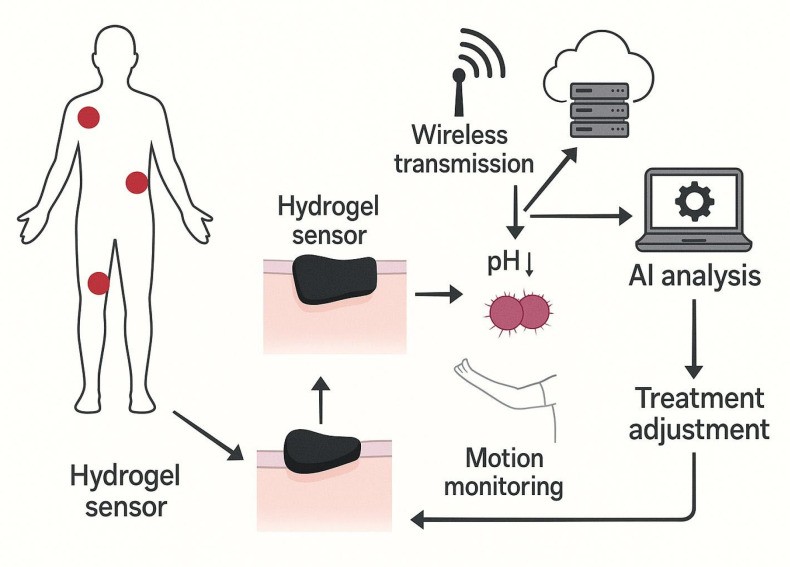
Monitoring systems for future treatment adjustment. Arrows denote the directional flow of system information: sensor placement and signal acquisition, pH-based infection detection, wireless data transmission to the analysis module, AI-driven processing, treatment-adjustment output, and the feedback loop for continuous motion and sensor monitoring.

During inflammation, injured tissues release exudate, a fluid rich in electrolytes, creatinine, fibrinogen, matrix metalloproteinases (MMPs), and key inflammatory proteins such as tumor necrosis factor-alpha (TNF-α), neutrophil gelatinase-associated lipocalin (NGAL), and C-reactive protein (CRP) [1,2,3,4]. TNF-α is a pivotal pro-inflammatory cytokine mediating local and systemic immune responses [2]. NGAL, a protein associated with neutrophil activity, is a biomarker for acute and chronic inflammation and is detectable in wound fluids [3]. CRP is a well-established systemic inflammation marker, elevated in wound exudate, reflecting the inflammatory status of tissues [29]. Detecting and monitoring these inflammatory proteins is essential for assessing wound progression and tailoring therapeutic interventions (Figure 9). A significant development is the creation of an electrochemical impedance spectroscopy (EIS)-based biosensor utilizing hyaluronic acid methacrylate (HAMA) hydrogel. This sensor demonstrated sensitive, real-time, and label-free detection of CRP in interstitial fluid, with a detection limit of 0.7 µg/mL in buffer and 0.8 µg/mL in artificial interstitial fluid. The biosensor required only 5 µL of sample and had a rapid response time of 100 s, showing excellent potential for wearable diagnostics and chronic wound monitoring [29]. Another advanced platform integrates molecularly imprinted polymers (MIPs) with natural HA chains to create a dual-responsive biosensor. This hybrid architecture combines the high specificity of MIPs with the natural affinity of HA, achieving selective CRP recognition under physiological conditions. The system displayed excellent sensitivity and selectivity toward CRP, opening avenues for hybrid hydrogel-based biosensors [29].

Although most HA-based biosensors target CRP, HA hydrogels have also been investigated for their interaction with other inflammatory mediators. For example, an injectable, self-healing HA hydrogel crosslinked via iron (Fe^3+^) coordination was developed to regulate matrix metalloproteinase-13 (MMP-13) activity, a critical enzyme involved in extracellular matrix degradation in chronic wounds. The HA-based structure allowed therapeutic and monitoring functions through its responsiveness to the inflammatory environment [29].

In addition, a dynamic HA hydrogel platform was engineered to respond to tumor necrosis factor-alpha (TNF-α), a major pro-inflammatory cytokine. The hydrogel matrix modulated cytokine interactions, demonstrating potential for biosensing and therapeutic release in chronic wound beds, although direct sensor readout was not the primary focus [28].

### 5.5. pH Detection

The wound healing process consists of three main stages: inflammation, proliferation, and remodeling. Healthy skin has a slightly acidic pH (4–6), which prevents bacterial growth. However, when an injury occurs, the pH rises to around 7.4, creating a favorable environment for bacterial proliferation and increasing the risk of infection [28]. Chronic wounds tend to be more alkaline (pH 7–9), making them more susceptible to bacterial colonization [28]. If the hydrogel degrades or loses mechanical integrity, bacterial proliferation can be assessed using both traditional microbiological assays (such as colony-forming unit counts or biofilm staining) and embedded biosensors. Electrochemical sensors can detect changes in impedance or open-circuit potential associated with bacterial activity, while optical sensors can monitor fluorescence or colorimetric shifts linked to bacterial metabolites. These approaches provide rapid infection detection, even before visible clinical signs appear [33]. Bacterial growth often increases wound pH towards alkaline levels, creating conditions that favor further microbial colonization and slow healing. HA hydrogels can be engineered to maintain a mildly acidic environment by incorporating pH buffers, releasing acidic compounds in response to pH changes, or embedding pH-responsive antimicrobial agents. Additionally, AI algorithms can continuously monitor pH fluctuations and recommend interventions, such as targeted antimicrobial release or dressing changes, when a threshold is reached [26,34,35].

Flexible optical pH sensors are effective tools for monitoring pH changes in wound environments. They use pH-sensitive dyes that change color based on pH levels. One challenge with this approach is ensuring the dyes adhere properly to wound dressings and respond to the appropriate pH range [35].

Trupp et al. developed an optical pH sensor using hydroxyl-substituted azobenzene dyes and cellulose films as a substrate. This sensor detects pH changes from 6 to 10 and can be laminated onto polyethylene terephthalate films to withstand mechanical stress and swelling. The sensor displays significant color changes from yellow (acidic) to red (basic), providing a visual pH map for wound healing. They also created wound dressing materials that change color to indicate healthy (yellow) or infected (purple) skin [28,30].

Liu et al. designed a pH-sensitive hydrogel wound patch using phenol red modified with methacrylate, copolymerized with an alginate/polyacrylamide matrix. This patch prevents dye leakage and offers high swelling capacity, mechanical strength, and a porous structure. Its color changes from yellow at pH 5–7 to orange at pH 7.4–8, and red at pH 9, making it suitable for monitoring chronic or infected wounds [28,31,36].

Electrospun nanofibrous films have also been developed for pH-sensitive wound dressings. These films offer a large surface area, high porosity, and better interaction with analytes, enhancing sensor sensitivity. A recent example includes electrospun films with curcumin-loaded polycaprolactone (PCL) matrices. Curcumin changes from yellow in acidic environments to red-brown in alkaline conditions (pH 6–9). A critical evaluation of the results indicated that this material enables visual pH detection without removing the dressing and allows easy wound condition assessment without medical training [28]. Despite their advantages, these optical pH sensors face a limitation in precisely quantifying pH levels, highlighting the need for more sensitive dyes for accurate measurement [37].

Wearable electrochemical pH sensors provide an accurate method for monitoring wound conditions by measuring electrical potential and current. Early sensors relied on glass electrodes, while modern versions used metals (Pd, Bi, Sb) and metal oxides (TiO_2_, ZnO). For enhanced flexibility and biocompatibility in wound monitoring, pH-sensitive polymers, including polyaniline (PANI) and polythiophenes have been developed [38].

Wang et al. [28,32] created a wearable electrochemical pH sensor integrated into a bandage, using electropolymerized PANI for pH detection. The sensor, fabricated via screen-printing, includes Ag/AgCl reference electrodes and carbon working electrodes, providing a stable Nernstian response of 59.2 mV/pH within the physiological range of 5.5–8. It also demonstrates strong elasticity and reproducibility [39].

Functionalization of HA-based hydrogels with integrated optical and electrochemical sensors has been developed due to the need to continuously monitor the wound environment and guide personalized treatment [28]. The sensors are incorporated into the hydrogel matrix to detect biomarkers relevant to the wound and its evolution. This approach is based on biocompatibility, high water retention capacity and hyaluronic acid (HA) regenerative properties, transforming a passive dressing into an active diagnostic tool [28].

## 6. Integration with AI and Wireless Monitoring

In the context of wound healing, AI plays a pivotal role in improving the reliability and accuracy of (bio)sensor data by handling signal noise and optimizing detection thresholds. Integrating artificial intelligence (AI) with HA-based hydrogel biosensors transforms raw sensor data into actionable clinical insights, revolutionizing wound care management [40]. This integration begins with continuous data acquisition, where biosensors embedded within the hydrogel matrix collect real-time information on key biomarkers such as pH, glucose, and inflammatory proteins from the wound microenvironment. The raw data undergoes preprocessing, consisting of filtering, smoothing, and normalization, to remove noise and artifacts, ensuring high data quality for reliable downstream analysis [40,41].

AI enhances sensor performance by directly improving signal interpretation and reliability. The effectiveness of AI and sensor integration is enhanced by the combination of advanced algorithms that compensate for sensor drift, enzymatic degradation, or environmental interference, effectively stabilizing long-term readings. Machine learning models can detect subtle biomarker changes that may be missed by standard thresholding, thereby increasing sensor sensitivity and enabling earlier detection of infection or delayed healing. Furthermore, AI can integrate multi-channel signals from functionalized HA hydrogels—such as pH-sensitive dyes, glucose oxidase reactions, or antibody-functionalised nanoparticles—optimizing signal extraction and reducing false positives caused by tissue movement or fluid variability [42,43,44].

By analyzing patient-specific trends, AI can provide tailored recommendations. For example, adaptive thresholding adjusts biomarker alert levels according to individual healing patterns, distinguishing normal inflammation from pathological changes. Spatial analyses with CNNs detect localized infections or necrotic tissue zones, while LSTM models track temporal biomarker fluctuations to differentiate transient inflammatory spikes from sustained infection or stalled healing. This ensures that clinicians receive actionable insights that directly inform dressing changes, therapeutic interventions, or antibiotic administration, rather than relying solely on raw sensor data.

Moreover, HA hydrogel properties enhance AI effectiveness: the hydrated, semi-permeable, and mechanically stable matrix provides consistent biomarker diffusion and signal integrity, allowing AI models to generate accurate, reliable predictions over time. For enzymatic glucose sensors, AI maintains precise readings for long-term patient monitoring [40,41].

A dedicated software architecture is essential to ensure seamless integration of AI with HA-based hydrogel biosensors in clinical or home environments. The software pipeline encompasses the full journey from data acquisition to clinical decision support. The system begins by interfacing with hydrogel sensors via Bluetooth Low Energy (BLE) or ZigBee, periodically sampling biomarker data and applying real-time filtering with timestamping and metadata tagging [45,46]. On-device firmware can perform preliminary signal conditioning and event detection, such as sudden pH drops, and encrypt the data prior to transmission. Once transmitted to a mobile device or cloud server, AI models—such as SVMs, CNNs, or LSTMs—process the data to classify healing phases, identify complications, and forecast wound outcomes. AI continuously evaluates sensor performance, dynamically calibrates thresholds, and enhances real-time detection accuracy, ensuring that even minor deviations in biomarker levels are detected reliably. Adaptive calibration modules within this platform learn patient-specific trends, improving precision over time. A user-friendly dashboard visualizes the analyzed information, offering graphs, tissue maps, and alert notifications. These outputs can be synchronized with electronic medical records (EMRs) via HL7 or FHIR protocols, ensuring clinicians have real-time access to patient data and can intervene promptly when necessary [45,46].

Real-world and literature-supported examples underscore the feasibility of AI integration in wound monitoring. For instance, Kim et al. (2022) demonstrated an AI-driven optical sensor patch capable of detecting pH and temperature changes in chronic wounds, with wireless data transmission to clinicians, significantly reducing infection-related hospitalisations [42,47]. Similarly, Li et al. (2023) integrated an electrochemical glucose sensor into a hydrogel wound dressing, coupled with a cloud-based AI system that predicted delayed healing with over 90% accuracy [42,48]. Commercial tools also exist: the Wound Viewer is an AI-powered medical device capable of 3D wound measurement and automated analysis uploaded directly into the EMR, while the ImitoWound app enables patients to photograph wounds using mobile devices, compute size, and track healing over time, validated through high intraclass correlation coefficients [33,40,49]. These cases demonstrate both the technical feasibility and clinical impact of AI-assisted HA hydrogel sensor platforms. (Figure 9).

## 7. Discussion

The reviewed results highlight the growing potential of hyaluronic acid (HA)-based hydrogel biosensors in the monitoring of chronic wounds. These smart platforms enable real-time, non-invasive detection of key biomarkers, such as pH, glucose, and inflammatory proteins. Their integration with optical and electrochemical technologies allows accurate wound assessment and early identification of infection risks, representing a significant advancement over conventional, visual-only monitoring techniques [2,3].

HA-functionalized biosensors add diagnostic and therapeutic value compared to earlier hydrogel systems used solely for maintaining moisture. The inclusion of biosensing components and AI-driven analytics transforms passive dressings into intelligent therapeutic tools. For instance, pH-sensitive sensors provide dynamic feedback on the inflammatory status, while glucose-sensitive patches enable better glycemic control in diabetic wounds [29].

A dual-responsive electrochemical biosensor was developed by integrating ultra-specific protein molecularly imprinted polymers (MIPs) and natural hyaluronic acid (HA) probes onto a flexible dual-channel screen-printed electrode (SPE) for the detection of the CD44 biomarker. MIPs were synthesized using alginate gel and CD44 as the template, while HA probes were immobilized on the second channel. The biosensor demonstrated high sensitivity and selectivity, with detection limits of 1.41 × 10^−5^ ng/mL (MIPs) and 1.51 ×10^−5^ ng/mL (HA), benefiting from the specificity, antifouling properties, and biocompatibility of both recognition elements, as well as the electrochemical stability of the SPE platform [36]. Hyaluronic acid (HA) and poly(ethylene glycol) (PEG) were co-immobilized via a one-step biomimetic surface functionalization, enabling enhanced recognition of soluble CD44. The combined antifouling properties of HA and PEG synergistically improved sensing specificity [45].

Considering the augmentation of effects of photobiomodulation for wound healing, an adhesive hyaluronic acid-based gelatin nanofibrous membrane integrated with multiple light-emitting diode (LED) arrays was developed as a skin-attachable patch [23,46].

From the perspective of previous studies, this convergence of biomaterials, biosensors, and AI reflects a broader trend in personalized medicine. Recent work by Fatima Mota et al. [5] and Mei Qin et al. [28] supports the notion that multifunctional biosensors embedded in flexible, biocompatible matrices can yield clinically actionable data without invasive procedures.

Future directions include developing fully self-powered biosensors, incorporating multi-biomarker detection in a single matrix, and integrating biosensor feedback with automated drug delivery systems. Cloud-based platforms using real-time sensor data and AI decision support may further enhance remote patient management and reduce healthcare burdens [48].

However, translating these technologies into routine care remains challenging. This includes ensuring the long-term biocompatibility and stability of HA hydrogels in vivo [18], addressing the power requirements for wearable sensors and wireless transmission, standardizing AI training datasets across diverse patient populations [49,50], and meeting regulatory criteria for data privacy and the ethical integration of AI in medical devices.

Conductive hydrogels (CHs) have become a key focus in developing smart wound dressings due to their unique properties that support both healing and monitoring. These materials support cell proliferation and migration by maintaining a stable structure and ensuring even current distribution across the wound [50]. However, they still face challenges, such as balancing conductivity and biocompatibility, avoiding immune responses, and adapting to complex wound environments [19].

Future research aims to improve CH design by making them more adaptive—able to dynamically respond to wound changes and optimize current conduction paths [51]. Integrating AI could further enhance their potential by enabling automatic adjustments based on the wound-healing stage and providing personalized treatments [52]. CHs combined with AI can provide precise sensing, monitoring, and data analysis, mimicking human senses like touch, smell, and vision.

Despite these advancements, several obstacles remain, including the collection of high-quality wound data, ensuring real-time monitoring, and maintaining stable skin contact without signal loss. Cloud-connected diagnostic platforms and multimodal sensing strategies could help manage the large, rapidly changing datasets generated by intelligent dressings. Developing flexible, low-power energy systems would also be essential to improve patient comfort and practicality [51].

Incorporating artificial intelligence (AI) significantly enhances the diagnostic and predictive capabilities of HA-based biosensors. AI-driven algorithms analyze real-time sensor data, identifying patterns that indicate infection risks, delayed healing, or other complications. These algorithms utilize machine learning models trained on historical patient data, thereby enhancing the accuracy of wound assessment and facilitating early interventions [51]. Berezo et al. developed a machine learning model capable of real-time prediction of the likelihood that chronic wounds will fail to heal by 4, 8, and 12 weeks after treatment initiation, based on assessments made at various time points [53].

**Figure 10:**
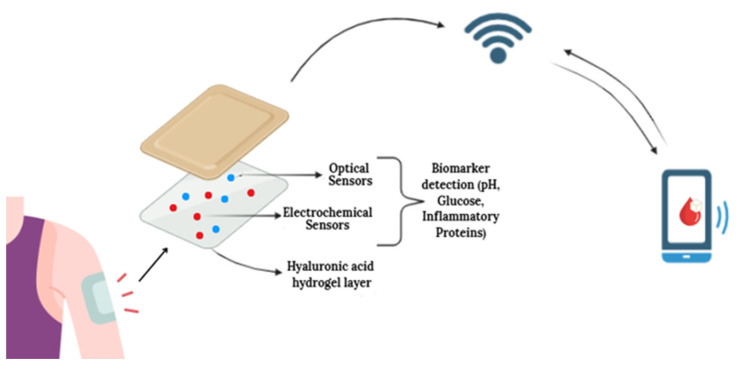
Schematic representation of intelligent biosensors based on hyaluronic acid hydrogels for monitoring chronic wound healing with the involvement of artificial intelligence. The patch on the skin allows interstitial fluid to reach the hyaluronic-acid hydrogel layer. The hydrogel delivers the collected biomarkers to the optical and electrochemical sensors. The sensor module sends the processed signals to the wireless communication unit. The wireless link and the mobile device are connected through a bidirectional channel. This indicates both data transmission from the sensor to the phone and potential commands or calibration signals sent from the phone back to the sensor. The mobile app displays the biomarker values in real time.

Wireless communication technologies further enhance the functionality of these biosensors by enabling remote monitoring (Figure 10). This feature allows healthcare providers to continuously track patient progress and adjust treatment protocols accordingly [25,54].

Zheng et al. [33] developed PETAL, a battery-free, paper-like AI-enabled sensor patch for real-time wound monitoring. It integrates five colorimetric sensors (pH, temperature, TMA, moisture, and uric acid) into a single paper patch, allowing rapid biomarker assessment within minutes. Using a deep learning algorithm and a smartphone, PETAL detects infection, inflammation, and wound conditions, classifies wound type and severity, and was validated both ex situ (with wound exudates) and in vivo (on rat models with chronic or burn wounds) [54].

Privacy and data security concerns are also significant, especially as AI-powered systems require access to sensitive medical information. The absence of a unified regulatory framework complicates addressing issues related to data ownership and transmission. Ensuring data protection and transparency will be crucial for maintaining trust and achieving positive outcomes in AI-driven medical care [6,55].

By integrating AI with biosensor networks, personalized treatment strategies can be developed, optimizing patient outcomes and reducing the burden of chronic wound management on healthcare systems. Additionally, the AI algorithms can control the release of HA, ensuring that the wound environment receives continuous support for healing based on real-time sensor feedback.

## 8. Conclusions

Chronic wounds (CWs) remain a significant global health challenge, affecting millions of patients and often leading to severe complications and diminished quality of life. The development of innovative biosensors based on hyaluronic acid (HA) hydrogels represents a promising advancement in wound management, enabling real-time, non-invasive monitoring of key biomarkers such as pH, glucose, and inflammatory proteins. Optical and electrochemical sensor technologies have shown significant progress, particularly in detecting biomarkers like pH and temperature, with several commercialized devices—such as Biohealth, VeCare, and Smartheal—demonstrating their practical applicability [6]. However, despite their clinical testing, these technologies are not yet widely implemented in routine practice due to production costs and scalability challenges.

Several factors must be addressed for these biosensing platforms to be successfully integrated into everyday medical practice. High sensitivity, biocompatibility, stability, and autonomous operation with wireless data transmission are important for widespread adoption. Moreover, considering that CWs are often fragile and require careful management, the materials used in these innovative dressings must be soft, non-aggressive, and comfortable for patients. Enhancing the mechanical and electrical conductivity of the sensors, as well as improving their durability and responsiveness, will be essential to their long-term success.

Integrating AI-driven data analysis with these biosensors further enhances their potential by enabling predictive analytics, early complication detection, and personalized treatment adjustments. These intelligent systems can optimize wound care strategies by continuously monitoring wound biomarkers in real time, allowing for timely interventions and improved healing outcomes [53,55].

In the future, the widespread adoption of HA-based hydrogel biosensors in wearable wound care devices has the potential to revolutionize personalized medicine. These advanced platforms will not only enhance wound healing and reduce complications but also provide clinicians and patients with precise, data-driven insights into wound management. As research continues to refine these technologies, their integration into routine healthcare settings will mark a transformative shift toward more efficient and effective chronic wound treatment.

## 9. Future Directions

Detection methods for chronic wound (CW) biomarkers face significant challenges due to the delayed diagnosis and difficulty in follow-up monitoring of wound healing. Traditional approaches rely on visual and physical examinations, patient medical history, and medication records. However, these methods are often subjective and require experienced professionals, making them ineffective for detecting underlying infected or dead tissues, which are critical for assessing wound healing. Conventional methods focus on identifying microorganisms at the wound site, as microbial presence is a key biomarker for CW diagnosis. Techniques like microbiological cultures are commonly used but have limitations, including the failure to identify non-culturable bacteria and the need for late-stage detection. Molecular assays, like rRNA-PCR and sequencing, provide more accurate results by detecting both cultivable and non-culturable bacteria, but they also face challenges like sample contamination and difficulty distinguishing active infections [23].

Advanced methods like electrochemical bioimaging and microscopy are also used, but they require specialized equipment and often produce false-negative results due to irregular bacterial distribution. Despite these efforts, current techniques often focus only on bacterial pathogens and lack a comprehensive view of the wound microenvironment, which includes multiple biomarkers like immune response indicators, growth factors, and cytokines [23].

Due to the high costs and inefficiency of current diagnostic approaches, there is an urgent need for innovative, accurate, and cost-effective detection methods. Recent research has explored sensor-based technologies for CW biomarker detection, providing real-time and reliable information on wound status. These sensors monitor changes in biomarker concentrations, such as enzyme levels and pH, which can indicate bacterial infection and delayed healing. Given the complexity and variability of the wound’s biological environment, developing sensors with high sensitivity, precision, and ease of use remains crucial for improving CW diagnosis and treatment outcomes [5,56].

Detection methods for chronic wound (CW) biomarkers face significant challenges due to the delayed diagnosis and difficulty in follow-up monitoring of wound healing. Traditional approaches rely on visual and physical examinations, patient medical history, and medication records. However, these methods are often subjective and require experienced professionals, making them ineffective for detecting underlying infected or dead tissues, which are critical for assessing wound healing [2,5].

While HA is relatively easy to functionalize, achieving consistent and reproducible results across large-scale production can be challenging. Surface and interfacial issues during manufacturing can also impact the (bio)sensor’s performance [23]. HA, like other biomaterials, may require stabilization techniques (e.g., with nanoparticles or conductive polymers) to prevent aggregation or degradation [51,56].

Despite significant advances, integrating AI technologies into routine clinical practice remains a complex challenge. The structural properties of the hydrogel enable it to conform closely to tissue surfaces, minimizing mechanical mismatch and promoting seamless integration with the host. This close integration is essential for continuous monitoring applications, ensuring long-term sensor stability and reliable performance [50,57]. Key issues include ensuring HA hydrogels’ long-term biocompatibility and in vivo stability, managing power demands for wearable sensors and wireless communication, standardizing AI training datasets across diverse patient populations, and adhering to regulatory standards for data privacy and the ethical deployment of AI in medical devices.

Future developments of hydrogels aim to create fully self-powered biosensors, enable simultaneous detection of multiple biomarkers within a single platform, and link biosensor feedback to automated drug delivery systems [58]. Additionally, cloud-based platforms that leverage real-time sensor data and AI-driven decision support could significantly improve remote patient monitoring and help alleviate pressures on healthcare systems.

## Figures and Tables

**Table 1:** Detection Parameters in HA-Based Biosensors for Chronic Wounds.

Parameter	Detection Method	Limit of Detection (LOD)	Sensitivity/Range	Reference
Glucose (electrochemical)	GOx-based amperometric detection in HA hydrogel	5 µM (standard) ~1 µM (with nanocomposites)	Linear up to 30 mM Rapid response (<5 min)	[3,28,29]
Glucose (optical)	Fluorescent boronic acid or protein-based probes	~0.1 mM	Effective in 0.1–25 mM range	[5,28,29]
H_2_O_2_ (ROS)	Electrochemical (Prussian Blue) or fluorescent probes	100 nM	Real-time monitoring of oxidative stress	[5,28]
Matrix metalloproteinase-9 (MMP-9)	Peptide-based fluorogenic or electrochemical sensors	5 ng/mL	Specific detection of proteolytic activity	[24,28]
C-reactive protein (CRP)	Electrochemical impedance spectroscopy (EIS) using HAMA (hyaluronic acid methacrylate)	0.7–0.8 µg/mL	Sample volume: 5 µL Response time: ~100 s	[29]
CRP (molecularly imprinted polymers MIP/HA)	MIP-HA hybrid biosensor	NS	High specificity/selectivity in physiological conditions	[29]
pH (optical)	pH-sensitive dyes (e.g., phenol red, curcumin, azobenzene)	pH range 5–10	Visual color shift (e.g., yellow to red)	[28,30,31]
pH (electrochemical)	PANI-based wearable pH sensor (screen-printed)	NS	59.2 mV/pH pH range: 5.5–8	[28,32]
